# Sulfated Polysaccharides in Marine Sponges: Extraction Methods and Anti-HIV Activity

**DOI:** 10.3390/md9010139

**Published:** 2011-01-24

**Authors:** Ana I. S. Esteves, Marisa Nicolai, Madalena Humanes, Joao Goncalves

**Affiliations:** 1 Centre of Marine Sciences, University of Algarve, Gambelas Campus, 8005-139 Faro, Portugal; 2 Oral and Biomedical Sciences Research Unit, Faculty of Dentistry, University of Lisbon, 1649-003 Lisbon, Portugal; E-Mail: mhnicolai@hotmail.com; 3 Centre of Chemistry and Biochemistry, Faculty of Sciences, University of Lisbon, Building C8, Campo Grande, 1749-016 Lisbon, Portugal; E-Mail: mmhumanes@fc.ul.pt; 4 Research Unit for Retrovirus and Associated Infections, Centre of Molecular Pathogenesis, Faculty of Farmacy, University of Lisbon, Avenida das Forcas Armadas, 1649-019 Lisbon, Portugal; E-Mail: joao.goncalves@ff.ul.pt

**Keywords:** porifera, *Erylus discophorus*, polysaccharides, anti-HIV activity

## Abstract

The extraction, fractionation and HIV-1 inhibition potential of polysaccharides extracted from three species of marine sponges, *Erylus discophorus*, *Cliona celata* and *Stelletta* sp., collected in the Northeastern Atlantic, is presented in this work. The anti-HIV activity of 23 polysaccharide pellets and three crude extracts was tested. Crude extracts prepared from *Erylus discophorus* specimens were all highly active against HIV-1 (90 to 95% inhibition). *Cliona celata* pellets showed low polysaccharide content (bellow 38.5%) and almost no anti-HIV activity (<10% inhibition). *Stelletta* sp. pellets, although quite rich in polysaccharide (up to 97.3%), showed only modest bioactivity (<36% HIV-1 inhibition). *Erylus discophorus* pellets were among the richest in terms of polysaccharide content (up to 98%) and the most active against HIV-1 (up to 95% inhibition). Chromatographic fractionation of the polysaccharide pellet obtained from a specimen of *Erylus discophorus* (B161) yielded only modestly active fractions. However, we could infer that the active molecule is most probably a high molecular weight sulfated polysaccharide (>2000 kDa), whose mechanism is possibly preventing viral attachment and entry (fusion inhibitor).

## 1. Introduction

Sponges are filter feeding benthonic animals that have survived until present almost morphologically unaltered since the Superior Cambrian (509 million years ago) [1]. They represent the simplest multicellular life form present nowadays in our planet, similar, in evolutionary terms, to primordial multicellular organisms [2]. Sponges are the most primitive animals living today and, for that, they are considered living fossils [3]. These animals are so peculiar that, in the Metazoa kingdom, a whole phylum is dedicated to them—Phylum Porifera [4]. Currently, there are 8365 known sponge species [5] and, of these, around 98% live in marine habitats [1]. They attach to the marine substratum in many different depths (from intertidal zones to abyssal pits), temperatures, salinities and light conditions [6] and exist in a multitude of colors and shapes.

Natural products have long been used in food, fragrances, pigments, insecticides, drugs, cosmetics, *etc.* The pioneering work of Bergmann and collaborators in the 50s, giving the first insight into the area of marine natural products, resulted in the discovery of nucleoside analogues synthesized by marine sponges [7,8]. These were isolated for the first time in the sponge *Cryptotethia crypta* and, years later, served as an inspiration for the synthesis of the first and best known antiretroviral compound: zidovudine or AZT [9].

In the late 80s, the National Cancer Institute set up a large-scale screening program, with a wide variety of marine invertebrates, terrestrial plants and microorganisms being tested for their anti-HIV activity [10,11]. In the following years, around 40.000 organic and aqueous extracts were tested and, surprisingly, around 15% demonstrated at least some HIV inhibitory effect [11]. Many of these extracts exhibited bioactivity profiles similar to sulfated dextran or other high molecular weight sulfated polysaccharides extracted from sponges and algae [10]. Since then, studies on the discovery and isolation of new natural substances, with unique structures, displaying anti-HIV activities, have proliferated, with compounds from many different origins being reported—terrestrial plants, algae, microorganisms (bacteria, fungi, microalgae), sponges, echinoderms, tunicates, corals, crustaceans, molluscs, *etc.*—and also covering a wide range of chemical varieties—peptides and proteins [12,13], glycoproteins [14], polysaccharides (anionic, sulfated), flavonoids, coumarines, terpenoids, alkaloids [15], polyphenols [16], sterols, sulfolipids, lactones, among others [9,11,17–19]. Among all marine organisms, sponges are the most prolific in novel compounds, with more than 200 new metabolites being reported every year, presenting biological activities as diverse as antibiotic, antitumor, antiviral and anti-inflammatory [20].

Despite the intense research effort by academic and corporate institutions, very few natural products with real potential have been identified or developed [21]. At present, there are 25 chemical compounds formally approved by the Food and Drug Administration for the treatment of human immunodeficiency virus infection [22], none of them is of natural origin.

Polysaccharides play a crucial role in cell interaction and recognition processes, participate in structural events and are associated with cell differentiation and/or proliferation processes [23,24]. Sulfated polysaccharides with unique structures are also present in several marine sponge species, taking part in cell adhesion and recognition processes [2,24–26]. Several extraction and characterization methods are known since 1947, as well as their anti-clotting and immunological properties [23]. Polysaccharides have also long been known for their antiviral properties [10]. Since 1988, the activity spectrum of the sulfated polysaccharides has been shown to extend to various enveloped viruses [27]. Against HIV, they possibly act by blocking the viral gp120 adsorption to the lymphocytic cell [28,29]. Although these polyanionic molecules exhibit a broad spectrum antiviral activity together with low viral resistance induction, their low specificity pharmacological properties lead to a weak anti-HIV activity *in vivo* [30].

In the marine environment, sulfated polysaccharides extracted from algae have been intensively studied for their antiretroviral activities [27,31–37] but only few examples regarding the antiviral activities of glycosidic molecules extracted from marine sponges have been reported in the literature [10,14,38,39]. In this work, we report preliminary assays on the anti-HIV activity of extracts from marine sponges collected in the Western Portuguese Coast, Northeastern Atlantic. *Stelletta* sp. (former *Myriastra* sp.), *Cliona celata* and *Erylus discophorus* anti-HIV surveys yielded a small activity for the first species, the second species produced almost no activity and only *E. discophorus* extracts presented an extremely potent inhibitory effect on HIV-1. Since polysaccharides can be responsible for this type of activity, we tested several polysaccharide fractionation methods and their anti-HIV potential.

## 2. Results and Discussion

### 2.1. Extraction Methods

Four different polysaccharide extraction methods were applied to six sponge samples: B22 (*Stelletta* sp.), B33 and B124 (*Cliona celata*) and B161, B206 and B294 (*Erylus discophorus*), thus providing us with 24 polysaccharide pellets (these pellets will be further addressed in the format specimen(extraction method); example: B33(II) would refer to the polysaccharide pellet extracted from specimen B33 according to extraction method II). B358 and B437 were only used in crude extract preparation; no polysaccharide extraction was performed with these samples.

**Figure 1:**
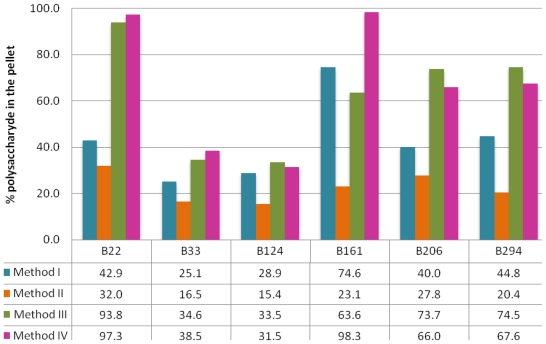
Percentage of sulfated polysaccharides in the final pellets ((polysaccharide mass determined by toluidine blue/total pellet weight) × 100).

Polysaccharide pellets were weighed once completely dry (Table 1), dissolved in water and assayed with toluidine blue for sulfated polysaccharide concentration (Table 2). Since there can be co-precipitation of other molecules in the pellet, percentage of sulfated polysaccharides effectively extracted in each pellet was calculated (Figure 1).

As can be seen in Tables 1 and 2, polysaccharide concentration varies largely among sponge samples and extraction methods. The polysaccharide content constitutes 0.02 to 0.39% of the total sponge dry weight for *Cliona celata* specimens, 0.66 to 22.3% for *Erylus discophorus* specimens and 0.67 to 4.2% for *Stelletta* sp. Several authors have reported variable contents of sulfated polysaccharides in marine sponges: Zierer *et al.* reported that, in four different marine sponge species, approximately 1% of the sponge dry weight consists of acidic polysaccharides [40,41]; Vilanova *et al.*, working with four different species of *Chondrilla* sp., reported a polysaccharide content of up to 3% of the sponge dry weight [42]. These results are probably a reflection of the high variability of polysaccharide content between different sponge species, not only in quantity but also in the type of polysaccharides, as reported by Vilanova *et al.* [42].

Looking at the results obtained for *Cliona celata* (B33 and B124), one can see that, regardless of extraction method, it is not only the species with lowest polysaccharide pellets (Table 1), ranging from 0.52 mg to 13.80 mg of polysaccharide pellet per g of dry sponge weight, but it also gave rise to the least rich polysaccharide pellets (Figure 1), with only 15.4 to 38.5% of the pellet being effectively composed of polysaccharides. *Erylus discophorus* seems to be the sponge species with highest content in terms of polysaccharide, with pellets weighing 20.80 to 299.20 mg per gram of sponge dry weight. *Erylus discophorus* specimens also provided some of the richest pellets in terms of polysaccharide percentage, with pellet B161(IV) being almost exclusively composed of polysaccharides (98.3%). Regarding *Stelletta* sp., it is difficult to extract general conclusions since only one specimen was analyzed. Even so, we can say that sample B22 would be a moderately rich sponge in terms of polysaccharide content and that extraction methods III and IV were particularly efficient, providing us with two pellets of very high polysaccharide yield (93.8 and 97.3% for B22(III) and B22(IV), respectively). To our knowledge, there are no previous studies on the polysaccharide content of these species, which does not allow us to further generalize our observations.

As can be seen in Figure 1, the least efficient method, in terms of polysaccharide extraction, is method II, followed by method I. Since the latter consists of a simple ethanolic precipitation, there is most likely co-precipitation of other cellular components such as proteins and nucleic acids, and thus low yields of polysaccharide concentration in the recovered pellets. Although method II seems to be somewhat more specific than method I, including an extraction step with CPC, polysaccharide content in method II pellets is consistently lower in all samples. One should take into account that, as previously reported by Beutler *et al.*, (1) the extraction methodology used in method I produces quite variable yields of polysaccharides and (2) the complexity of the matrix may influence the accuracy of the colorimetric determination using toluidine blue.

Extraction methods III and IV were the most efficient, with a content of up to 98.3% polysaccharides in the pellet obtained from sample B161 using method IV. Since extraction methods III and IV are more complex, involving further fractionation steps, these results are not surprising. The fractionation steps of proteolytic digestion with papain and nucleic acid degradation with DNase that comprise method IV, clearly made a difference when regarding polysaccharide purity, giving rise to two of the richest pellets in terms of polysaccharide content: B22(IV) and B161(IV), with a content of 97.3% and 98.3%, respectively. Thus, method IV would obviously be the preferred method for polysaccharide extraction in these samples.

### 2.2. HIV-1 Inhibition Activity

All polysaccharide pellets (except for B33(IV) which was lost) were dissolved in double distilled water, in a 50 mg/mL proportion, and tested for their anti-HIV properties. Crude extracts from B161 and two other *Erylus discophorus* samples, B358 and B437, were also assayed for anti-HIV activity. All samples were checked for cell viability, discarded in case of massive cell death and assayed again. P24 concentrations were extrapolated in a previously constructed p24 standard titration curve and inhibition percentage was calculated after setting the positive control as 0% inhibition and negative control as 100% inhibition.

**Figure 2:**
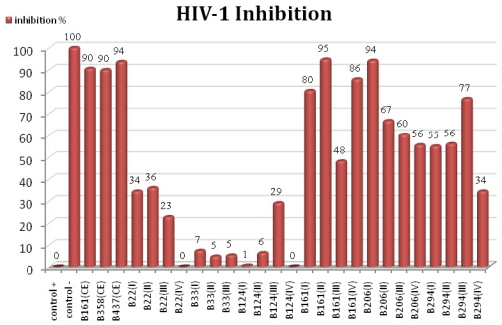
HIV-1 inhibition activity of crude extracts and polysaccharide pellets prepared from marine sponge samples belonging to three different species: *Erylus discophorus* (B161, B358, B437, B206 and B294), *Stelletta* sp. (B22) and *Cliona celata* (B33 and B124); CE: crude extract; I, II, III and IV refer to polysaccharide extraction method.

As can be seen in Figure 2, all crude extracts prepared from *Erylus discophorus* specimens presented an extremely potent inhibitory activity (90 to 95% HIV-1 inhibition). Regarding polysaccharide pellets, although pellets obtained from sample B22 were richer in terms of polysaccharide percentage, they present only moderate HIV inhibition as opposed to *Erylus discophorus* specimens, in which almost all polysaccharide pellets, regardless of specimen or extraction method, presented an HIV inhibition percentage of over 50%, exceptions being B161(III) and B294(IV). This observation points to the presence of polysaccharides with different structural features in marine sponges belonging to *Erylus discophorus* species. Indeed, there are several studies reporting the direct correlation of anti-HIV activity of sulfated polysaccharides with specific structural parameters [33], such as: increasing molecular weight and degree of sulfation, homopolymerization [27,35], charge density [43] and nature of counter-ion [44].

Sponge sample B161 originated three of the most HIV-inhibitory pellets: B161(I), B161(II) and B161(IV) with 80, 95 and 86% inhibition percentage, respectively. Given that the B161(IV) pellet is almost exclusively constituted of sulfated polysaccharides (98.3%), they are most likely responsible for the observed HIV-inhibitory activity. Although the B161(II) pellet showed the highest inhibition percentage, its polysaccharide content is quite low, probably due to few fractionation steps in extraction method II.

Polysaccharide pellets obtained from *Cliona celata* specimens presented the lowest inhibitory effect (lower than 10%, except for B124(III)), which can be correlated with low polysaccharide content.

B161(CE) test for the drug susceptibility assay performed in pre-infected cells showed no inhibitory effect of the tested sample, implying that the molecule in study affects infection probably by preventing HIV attachment and entry into the lymphocytic cell, working as a fusion inhibitor. These observations are also in accordance with previous studies showing the involvement of sulfated polysaccharides in blocking early events of viral replication [27,31,43,45,46].

### 2.3. Bioassay Guided Fractionation

**Figure 3:**
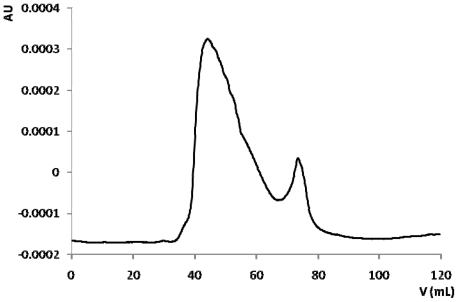
B161(IV) gel filtration chromatography in Sephacryl S-300 HR.

B161 was chosen for further bioassay guided fractionation of the polysaccharide pellet due to several reasons: high available biomass, high inhibition activity against HIV and rich polysaccharide pellets. The polysaccharide pellet obtained from sponge sample B161 with extraction method IV, B161(IV), was dissolved in double distilled water and fractionated in a previously calibrated gel filtration chromatography column, resulting in the chromatogram presented in Figure 3.

Fractions corresponding to each of the two peaks observed were pooled and concentrated. Concentrated fractions were tested for HIV inhibition and activity remained exclusively in the first peak. Elution volume corresponding to maximum absorbance at 280 nm was around 45 mL. Elution volume of dextrane blue (2000 kDa molecular weight), as determined in the column calibration (results not shown), was around 52 mL. Thus, according to these data, the biologically active molecule has a very high molecular weight of over 2000 kDa. Previous studies on anti-HIV sulfated polysaccharides extracted from marine invertebrates are consistent, reporting molecular weights of 500 to 1000 kDa [10] and over 1500 kDa with high degree of sulfation [39].

The fact that the active fraction absorbs at 280 nm may indicate the presence of proteic components. Whether these are contaminants or comprise an active glycoprotein or proteoglycan, remains unknown. Sponge proteoglycans, otherwise known as aggregation factors, are large molecules with approximate molecular weights ranging from 2 × 10^4^ kDa to 1.4 × 10^6^ kDa [26]. There are no previous records of anti-HIV proteoglycans in marine sponges but a few bioactive protein and glycoproteins have been reported [12,14].

Although this chromatography was successful in giving us some insights into a few molecular characteristics, it did not constitute a good separation step, since there was massive bioactivity loss. Several other chromatographic separations were attempted, none with satisfactory results regarding fractionation and bioactivity maintenance. The huge loss of bioactivity observed can be due to several problems: (1) a concentration problem in chromatography fractions; (2) HIV-1 inhibition activity results from the synergistic effect of more than one molecule, hence the activity loss when the sample is fractionated; (3) fractionation and consequent dilution results in loss of stability of the bioactive molecule; (4) bioactivity is merely due to a stereochemical hindrance effect of the bioactive molecule on the lymphocytic cell receptors, preventing HIV to attach and initiate subsequent infection events.

## 3. Experimental Section

### 3.1. Biological Material

Samples were collected by scuba diving at Reserva Natural da Berlenga (Portuguese western coast, Northeastern Atlantic) in several missions (1998–2004) at depths of 4–10 m. Immediately after collection, a voucher sample was taken in ethanol 90% for identification and the remaining sample was frozen at −20 °C, transported to the laboratory in isothermal containers and stored at this temperature until required.

All samples had an identification number. Table 3 summarizes sponge samples used in this study: five specimens of *Erylus discophorus* (B161, B206, B294, B358 and B437), two specimens of *Cliona celata* (B33, B124) and one specimen of *Stelletta* sp. (B22), all collected in Reserva Natural da Berlenga.

### 3.2. Crude Extract Preparation

Around 65 g of tissue from each sponge specimen was cut into small pieces, ground and homogenized in double distilled water (10% w/v). The resulting homogenate was stirred for 30 min at 4 °C, followed by centrifugation (15,300 g for 35 min at 4 °C). The pellet was discarded and the supernatant consisted of the crude extract (CE).

### 3.3. Polysaccharide Extraction

Four different procedures were used to extract polysaccharides from the biological material:

Method I [10]—An equal volume of ethanol was added (*ca.* 650 mL) to the previously prepared crude extract. This ethanol suspension was left overnight at −20 °C. The precipitate was separated by centrifugation (3840 g for 60 min at 4 °C) and dried in an oven at 60 °C.

Method II [47]—The crude extract was heated at 37 °C in a water bath, together with a solution of 5% (w/v) of cetylpyridinium chloride (CPC). The addition of one volume of the CPC solution, dropwise, to 5 volumes of the extract yielded a floccular precipitate. When all the solution had been added, the mixture was left for one more hour to ensure complete precipitation of the polysaccharides. These were separated by centrifugation (3840 g for 5 min at room temperature). The precipitate was dissolved in 2 M NaCl-ethanol (100:15, v/v) to convert the cetylpyridinium complexes into sodium salts (1 volume of this solution to 5 volumes of the aqueous extract).

The polysaccharides were precipitated from this solution using 3 volumes of ethanol. The mixture was left in an ice bath for 1 h, prior to centrifugation (15,300 g for 35 min at room temperature). The pellet was again dissolved in double distilled water. The final precipitate formed after the addition of 3 volumes of ethanol and incubation overnight at 4 °C. The precipitate, collected by centrifugation (15,300 g for 35 min at room temperature), was dried in an oven at 60 °C.

Method III [48]—A piece of sponge was thawed and added to *circa* 10 volumes of acetone for 24 h at 4 °C, after which it was chopped into small pieces and dried in an oven at 60 °C. The dried tissue (10 g) was suspended in 300 mL of 0.1 M sodium acetate buffer (pH 5.0), containing 1 g of papain, 5 mM cystein and 5 mM EDTA. This suspension was incubated at 60 °C for 24 h. After centrifugation (2460 g for 15 min at 10 °C), the polysaccharides present in the supernatant were precipitated with 16 mL of a 10% (w/v) solution of cetylpyridinium chloride (CPC). The mixture was left for 24 h at room temperature, and centrifuged (2460 g for 15 min at room temperature). The precipitated polysaccharides were resuspended in 150 mL of 2 M NaCl-ethanol (100:15, v/v) and again precipitated with 300 mL ethanol 95%. After 24 h at 4 °C, the precipitate was collected by centrifugation and washed twice with 300 mL 80% ethanol and once with 95% ethanol. The final product was dried in an oven at 60 °C.

Method IV [40]—A piece of sponge was thawed and added to *circa* 10 volumes of acetone for 24 h at 4 °C. After this time, it was chopped into small pieces and dried in an oven at 60 °C. The dried tissue (10 g) was suspended in 300 mL of 0.1 M sodium acetate buffer (pH 5.0), containing 1 g of papain, 5 mM cystein and 5 mM EDTA. This suspension was incubated at 60 °C for 24 h and centrifuged (2460 g for 15 min at 10 °C). The supernatant was heated at 100 °C for 10 min and cooled to room temperature. Then, it was incubated with 1060 U DNase I for 24 h at room temperature, with stiring. Centrifugation (24,000 g for 15 min at 10 °C) yielded a supernatant that was precipitated with 37.5 mL of 10% (w/v) CPC. The mixture was left for 24 h at room temperature and centrifuged again (2460 g for 15 min at room temperature). The precipitated polysaccharides were resuspended in 150 mL of 2 M NaCl-ethanol (100:15 v/v) and again precipitated with 300 mL ethanol 95%. After 24 h at 4 °C, the precipitate was collected by centrifugation and washed twice with 300 mL of 80% ethanol and once with 95% ethanol. The final product was dried in an oven at 60 °C.

### 3.4. Quantification of Sulfated Polysaccharides

Sulfated polysaccharides were quantified using the toluidine blue assay [10,49] and fucoidan as standard. Polysaccharide pellets obtained from each extraction were dissolved in double distilled water and diluted until polysaccharide concentration was in the range of the calibration curve and could be calculated by direct extrapolation. 200 μL aliquots of standard fucoidan solutions, in the range of 0 to 10 μg, and diluted samples were mixed with 1 mL of a 0.01 mg/mL solution of toluidine blue and, after 10 min, absorbance readings were taken at 620 nm. Polysaccharide determinations in both standards and samples were made in triplicate and median values were used for calculations.

### 3.5. Chromatographic Fractionation

Chromatographies were performed in a FPLC (Fast Protein Liquid Chromatography) system, coupled to a UV detector set at 280 nm and an automated fraction collector (Pharmacia Biotech). Sponge sample B161 was chosen for subsequent fractionation due to higher available biomass together with very active polysaccharide pellet.

The final polysaccharide pellet obtained from sample B161 with extraction method IV, B161(IV), was dissolved in double distilled water in 50 mg/mL proportion. This solution was fractionated using gel filtration chromatography (Sephacryl S-300 High Resolution, Pharmacia Biotech), in a manually packed column of 140 mL bed volume. Sample (2 mL) was eluted with 100 mL of double distilled water, at a constant flow rate of 0.5 mL/min.

Fractions corresponding to each chromatographic peak were pooled, concentrated (using ultrafiltration) and tested for HIV-1 inhibition activity.

### 3.6. HIV-1 Inhibition Determination

HIV-1 inhibition activity was determined using the *in vitro* HIV drug susceptibility assay [50], which measures the extent to which a drug inhibits HIV p24 antigen production by lymphocytic cells acutely infected with a viral isolate. The HIV-1 inoculum, NL4-3 strain, was produced by transfection of 293T lymphoblastic cells, using Fugene 6 (Roche) as the transfection agent. The viral supernatant was collected 48 h after transfection, aliquoted and kept at −20 °C until required. Viral infectivity, expressed in terms of 50% Tissue Culture Infectious Dose (TCID_50_), was determined prior to the drug susceptibility test, using a streamlined endpoint dilution assay that was analyzed by the Spearman-Karber statistical method [50]. Both infectivity and drug susceptibility assays were performed using Jurkat lymphocytic cell line.

The drug susceptibility assay was performed in 96-well plates using 200,000 Jurkat cells per well, 1000 TCID_50_ of the viral inoculum per million cells, 20 μL of sample (or sample buffer in the case of the positive control) and cell culture medium to 200 μL volume completion. Negative control consisted of non-infected cells and sample buffer. Prior to infection, cells were incubated with each sample for 1 h at 37 °C, after which the HIV inoculum was added. On day 4, cells were examined microscopically and 125 μL of cell suspension from each well was mixed, removed and discarded, followed by new addition of 20 μL of sample and 130 μL of growth medium. On day 7, HIV p24 antigen concentration was determined using the INNOTEST HIV Antigen mAb kit (Innogenetics), according to the manufacturer’s instructions. For a preliminary insight into the viral inhibition mechanism, an assay was performed with pre-infected cells incubated with a previously known inhibitory sample.

In parallel, all samples were checked for cell viability, serving as a control for the drug susceptibility assay, making sure all inhibitory effects would be due to the sample itself and not to cell death (implicating low or none viral replication). The cell viability assay replicated the drug susceptibility assay but with no viral inoculum. On day 7, 20 μL of the cell proliferation reagent WST-1 was added to each well, incubated for 30 min to 4 h and absorbance readings at 450 nm were taken, with a reference filter of 620 nm.

## 4. Conclusions

AIDS and HIV infection is one of the most worrying world epidemics nowadays, with consequences at political, social, economic, scientific and medical levels. Despite all efforts in fighting this disease, it does not show signs of slowing down [51]. There is an urge for new and more efficient drugs, able to overcome viral resistance, with lower side effects to the patient and the ability to provide total viral clearance. In this context, the scientific community has joined efforts in the research and development of novel compounds capable of permanently eradicating this disease.

More than often, nature has served as inspiration or source for new molecules with extremely potent antiviral properties. With the rise and access to new submersion technologies, researchers have turned their attention to the marine environment in the quest for finding new promising bioactive compounds. Knowledge has been gained in unique ecosystems, extraordinary organisms with noteworthy adaptations and exclusive compounds demonstrating a wide range of biological and pharmacological properties. Marine sponges, in this matter, were revealed to be important sources of new substances and interesting research targets in biotechnology [52].

In this work, we have discovered and explored the extremely potent HIV-1 inhibitory activity present in the marine sponge *Erylus discophorus*. This bioactivity seems to be species-specific, in the matter that it is consistently found in all *Erylus discophorus* specimens. The molecule responsible for the anti-HIV activity is most probably a high molecular weight sulfated polysaccharide (over 2000 kDa). Inhibition mechanism is expected to work at the viral entry, preventing HIV adsorption and fusion with the lymphocytic cell.

Over the last 20 years of microbicide research, none of the 11 effectiveness trials of six candidate products have demonstrated meaningful protection against HIV infection [53]. Although the systemic applications of sulfated polysaccharides may have many drawbacks, their structure and mode of action indicate potential for topical uses to prevent virus infection [21]; they are able to block HIV without toxicity to the host cells, they are known to lead very slowly to virus drug resistance development and they show activity against HIV mutants that have become resistant to reverse transcriptase inhibitors [27]. Even if isolation of the bioactive compound is not feasible, the broad spectrum anti-HIV activity of the polysaccharide extracts from *Erylus discophorus* samples could be of use as a first antiviral barrier if applicable in vaginal gels or lubricants, should it prove to be non-toxic and effective *in vivo*.

## Figures and Tables

**Table 1:** Dry weight (mg) of the final polysaccharide pellets obtained per gram of dry sponge tissue.

Method	I	II	III	IV
Sample				
B22	98.09	20.87	35.47	24.07
B33	9.43	13.80	5.46	0.52
B124	13.37	8.39	6.22	4.72
B161	299.20	84.27	89.44	35.59
B206	139.46	28.36	81.08	20.80
B294	135.15	32.27	52.02	20.76

**Table 2:** Sulfated polysaccharide quantification as determined by the toluidine blue assay (mg of sulfated polysaccharide per gram of dry sponge tissue).

Method	I	II	III	IV
Sample				
B22	42.11	6.67	33.27	23.41
B33	2.36	2.28	1.89	0.20
B124	3.86	1.29	2.09	1.49
B161	223.34	19.46	56.84	34.98
B206	55.72	7.90	59.72	13.72
B294	60.57	6.59	38.76	14.03

**Table 3:** Sponge samples and species used in this study.

Sample	Species
B161	*Erylus discophorus*
B358	
B437	
B206	
B294	

B22	*Stelletta* sp.

B33	*Cliona celata*
B124	
